# Response to Goldstein et al. (2022) Humanitarian action in academic institutions: a case study in the ethical stewardship of unidentified forensic cases

**DOI:** 10.1080/20961790.2022.2107973

**Published:** 2022-10-06

**Authors:** Nicholas Passalacqua, Brian Spatola, Richard Thomas

**Affiliations:** a Department of Anthropology, Western Carolina University, Cullowhee, NC, USA; b National Museum of Health and Medicine, Silver Spring, MD, USA; c Anthropology Unit, Federal Bureau of Investigation, Quantico, VA, USA

Dear editor,

In opening, we want to thank Goldstein et al. [1] and acknowledge their hard work and dedication to the practice of forensic anthropology and to their work identifying unknown remains. We agree wholeheartedly that there is a long-standing need for innovative and collaborative approaches to unidentified, cold-case investigations and their article is an important contribution to that literature [2–6]. However, we believe there are overarching legal and administrative issues that must be considered, and that the solution proposed in their manuscript for how academic institutions can “act as long term stewards of the unidentified” [1], omits any discussion of the critical failures of the overarching medicolegal authorities involved and the need for rigorous quality assurance protocols within academic laboratories interested in such an engaged level of involvement in the medicolegal system.

As Goldstein et al. [1] and others have pointed out, unidentified and missing persons cases in the US are a humanitarian crisis, sometimes referred to as a “silent mass disaster” [7]. We fully agree that in many circumstances unidentified individuals represent a burden on the medicolegal authority, which for them is unfortunately most easily handled *via* cremation or state-funded burial, and which may or may not be documented in a way that allows for later exhumation and re-analysis [8]. We argue that the primary issues discussed by Goldstein and colleagues are the result of a lack of sufficient resources to the medicolegal death investigation system to properly retain evidence (i.e. human remains) and to thoroughly (re)investigate unidentified “cold” cases.

The medicolegal death investigation system in the US is grossly underfunded [9–11]. This lack of appropriate resources results in numerous negative outcomes, including non-competitive pay [12,13], overwork, burnout [14–16], and insufficient time to investigate active and cold cases [17–19]. The solution proposed by Goldstein et al. [1] is essentially that the failings of the medicolegal death investigation system become the responsibility of academic forensic anthropology programmes.

Forensic anthropologists in academic and consulting roles do frequently find themselves deeply involved in the difficulties of medicolegal authorities maintaining proper curation and safeguarding of skeletal evidence, case data, and records of “forgotten” forensic cases. However, when anthropologists reflexively absorb the roles and responsibilities of medicolegal agencies who are legally mandated and authorized to carry out these duties, the inherent problems within the overarching system are frequently not considered or thoroughly addressed. We believe the situation outlined by Goldstein and colleagues is a familiar one to anthropologists throughout the US and that at the very least, the approach outlined in their paper should be part of a significant effort to establish the formal responsibilities of each submitting agency (if determinable) and the curating institution in accordance with local, state, and federal laws.

Goldstein and colleagues note that prior to establishing their current case file documentation procedures, their own laboratory had inconsistent numbering, missing recovery reports, missing casefile information, incomplete data collection sheets (i.e. analytical notes), inconsistent DNA collection, inconsistent submission to NamUS, and in some cases, total loss of case context [1]. While these circumstances are largely outside of the control of the current anthropologist(s) trying to improve case management, these examples are all serious quality assurance failures and unfortunately are not uncommon problems plaguing many unidentified remains cases in the US.

Goldstein et al. [1] propose a model for academic forensic anthropology programmes to follow should they want to accept unidentified forensic cases for long-term curation on behalf of a medicolegal authority in order to address these issues. The justification for this proposition is that academic curation of unidentified remains is “preferable” to the alternative of “cremation or inconsistently documented burials, both of which may severely limit the potential avenues for a positive identification” [1]. The authors note that long-term curation of forensic cases at academic institutions presents a number of challenges and that “rather than accepting cases for long-term storage without hesitation, academic institutions should consider whether accepting cases is ethical based on the availability of physical resources (e.g. laboratory and/or curation space, operating budgets) and time investments that practitioners in academia can feasibly commit towards the analysis, identification, and active stewardship of remains. In scenarios where this is not feasible, exploring collaborative options with other institutions may be beneficial to the analyst and identification of the unidentified individual alike” [1]. This latter statement is important and points to the fact that curation of active medicolegal cases should not be taken lightly. While we applaud the positive steps the processes outlined in the article calling to properly manage these cases, Goldstein et al. do not directly address what is needed for programmes with this level of involvement in medicolegal casework: that is, a fully functioning quality assurance (QA) programme with complete traceability and established protocols developed under the guidance of ISO 17025 or 17020 standards, ideally with accreditation of the anthropology laboratory as the ultimate goal. In fact, adherence to ISO standards and regular accreditation assessments would likely go a long way to resolving many of the problems previously mentioned with regards to unidentified remains cases.

Unfortunately, the vast majority of academic anthropology programmes (and public colleges and universities in general) are also understaffed, under-funded, and lacking in resources, to include space and time to devote to the analysis of unidentified forensic cases (which typically do not significantly count toward the three pillars of tenure: teaching, scholarship, or service) [20,21]. It is generally not part of the mission of colleges and universities to undertake medicolegal investigations, and as turnover occurs within anthropology departments (as mentioned in the article), subsequent academicians may not have the prerequisite knowledge, ability, or even the interest in properly continuing with any unidentified remains investigations curated by the previous faculty. More importantly, it is likely that the level of institutional support provided to the authors for medicolegal casework is extremely rare in anthropology departments nationwide. As a result, we believe that while the programme outlined by Goldstein et al. contains useful guidelines for curation of human remains cases, it is not an approach that is likely to be successful at other universities with less institutional support, and if adopted, would likely lead to future failures, similar to those mentioned by Goldstein et al. when faculty at Texas State assumed responsibility for the unidentified cases at their institution.

With regard to forensic anthropologists as experts in human identification, we agree that forensic anthropologists may be the professionals with the most appropriate expertise to investigate unidentified cold cases, *if they represent human skeletal remains*. While data on the condition of unidentified remains are not readily available, we suggest that many, if not the majority, of unidentified individuals that become cold cases do not enter the medicolegal death investigation system as skeletonized remains, and in such circumstances, forensic anthropologists may not be readily considered by medicolegal staff as necessary in the investigation or may not have the appropriate facilities to process large numbers of human bodies into skeletal remains for long-term curation.

Additionally, the authors state “forensic anthropologists are often responsible for the management of long-term unidentified individuals” [1]. While academic forensic anthropologists often retain forensic cases long-term after the conclusion of their analyses, we argue that this is generally inappropriate unless there is an established legal agreement between the institution (not a faculty member) and the medicolegal authority that ensures that the ultimate responsibility for ongoing investigation and disposition clearly lies with the proper authority. The retaining of medicolegal evidence by academic departments or museums requires a good faith effort to establish agreements for moving forward and retroactively for any remains from the past, in order to ensure all parties are clearly acting within the sphere of their respective legal and ethical responsibilities. We argue this is critical for highlighting issues related to funding and any lack of resources generally felt within a given medicolegal authority, and conversely is a way for well-funded authorities to improve their standard operating procedures and the level of collaboration with academic forensic anthropologists.

Unidentified individuals represent active forensic cases, and as such, they fall under the purview of the medicolegal authority. By retaining forensic cases after the completion of work, well-intentioned forensic anthropologists may be preventing additional investigation on the part of the medicolegal authority, and/or violating the next of kin’s right to sepulcher [22,23]. Rather, we suggest that if academic forensic anthropologists believe they would be better long-term stewards of unidentified remains than the medicolegal authority responsible, that they work with that medicolegal authority to generate some formalized cooperative agreement, possibly through a memorandum of understanding (MOU) or memorandum of agreement (MOA), as appropriate. These agreements should go beyond the simple “permission for curation” document provided by Goldstein et al. and specifically detail the terms of the agreement and expectations for both parties. The roles and liability for both parties must be clearly stipulated.

Additionally, we argue that the authors fail to properly articulate the fact that by taking custody of active forensic cases in the form of unidentified remains, they are accepting legal and financial liability for these cases. The proposition that academic forensic anthropology programmes act as long-term curators brings a number of issues, not just in terms of resources, but also in terms of long-term management of chain of custody, contact with external stakeholders (including next of kin to facilitate an identification and/or return the remains), and the potential for the misuse of evidence as teaching or research specimens. All these possible issues were recently exemplified in the scandal involving the conduct of doctors Mann and Monge and the remains of a victim from the 1985 MOVE bombing. In that case, these individuals retained unidentified remains of an active forensic case for over 30 years, never attempting to return them to the medicolegal authority, showed them to numerous individuals as part of museum tours, used them as teaching specimens without consent, and directly contacted the next of kin in attempt to make an identification outside of the medicolegal death investigation system [23].

We believe the most important point made by Goldstein and colleagues is that forensic anthropologists can be better advocates for the treatment of the unidentified by addressing the legacy of administrative gaps left by an overburdened or improperly managed medicolegal system. However, we propose that rather than taking on the legal, financial, and ethical responsibilities of the medicolegal authority, forensic anthropologists should work collaboratively with the medicolegal authority to advocate for more resources, including application for a wide variety of available federal grants, in order to support the medicolegal authority in their work and assist with their effort to make necessary systemic changes within the appropriate governmental authority.

Anthropological consultation will always require acceptance of some proportion of legal and ethical responsibility. The need for this acceptance of liability is due in part to the frequently insufficient nature of the medicolegal death investigation system of the US. Otherwise, there would be no need for outside consultation at all. However, we believe that the model proposed by Goldstein et al. [1] does not include the necessary legal administrative procedures or quality assurance standards to properly curate unidentified remains cases, and will, in many circumstances, require academic forensic anthropologists to assume more responsibility than they envision and more liability than their university is prepared to accept.

Finally, looking forward, we suggest that rather than having academic forensic anthropology programmes develop large QA systems and assume responsibility and liability for medicolegal authorities, that perhaps a better model would be for the development of permanent satellite human remains evidence storage facilities in large jurisdictions, states, or even at the federal level.


Nicholas Passalacqua
Department of Anthropology, Western Carolina University, Cullowhee, NC, USA nvpassalacqua@wcu.edu

Brian Spatola
National Museum of Health and Medicine, Silver Spring, MD, USA

Richard Thomas
Anthropology Unit, Federal Bureau of Investigation, Quantico, VA, USA


